# Characterization of the complete mitochondrial genome of *Thyonella gemmata* (Echinodermata: Cucumariidae)

**DOI:** 10.1080/23802359.2021.1975512

**Published:** 2021-09-20

**Authors:** Alexia C. Figueroa, William J. McHugh, Shane M. Miller, Amy K. Fellgren, Viktoria E. Bogantes, Alexis M. Janosik

**Affiliations:** University of West Florida, Pensacola, FL, USA

**Keywords:** *Thyonella gemmata*, Green sea cucumber, Holothuroidea, mitogenome, mtDNA

## Abstract

*Thyonella gemmata* , also known as the Green sea cucumber, is a biomedically and ecologically important species. In this study, the complete mitogenome of *T. gemmata* (Echinodermata: Holothuroidea) collected from the Florida Panhandle, USA is reported. The mitochondrial genome of *T. gemmata* consisted of 15,696 base pairs, and was composed of 36.10% A, 28.27% T, 23.18% C, and 12.45% G. There were 13 protein coding genes, 22 tRNA genes, and 2 rRNA genes within the mitogenome of *T. gemmata.* Mapping out the complete mitochondrial genome of *T. gemmata,* will help aid in future evolutionary studies and can be applied to future phylogenetic research of holothurians and related species.

Sea cucumbers, especially those that are members of coral reef ecosystems, play crucial roles in marine ecosystems. Specifically, the Green sea cucumber, *Thyonella gemmata* (Pourtalès 1851), influences the surrounding ecosystem through deposit-feeding of sediment organic matter (Marrugo-Negrete 2021). When *T. gemmata* digests sand, it releases calcium carbonate to the surrounding water (i.e. bioturbation), which aids in and promotes the growth of coral reef ecosystems (Pourtalès 1851; Uthicke 1999). Furthermore, *T. gemmata* is a bioindicator species, as its presence indicates a high level of biodiversity in subtropical and tropical ecosystems (Marrugo-Negrete 2021). *Thyonella gemmata* is primarily distributed in the shallow waters of the Atlantic Ocean with some occurrences off the West Coast of the United States throughout the Pacific Ocean, with a depth range of 0−6 m (Pawson et al. 2010). *Thyonella gemmata* population diversity can be recorded through sequencing of the mitochondrial DNA as a method of genomic identification, which is more accurate than employing morphological differences between species (Pawson et al. 2010). The complete mitogenome of *T. gemmata* reported here will provide information for future genetic research.

A tissue sample of *T. gemmata* was collected off the coast of Lower Grand Lagoon Bay, FL (30°07′25.1″N, 85°43′57.8″W), preserved in 200 proof ethanol, and deposited at the Florida Museum of Natural History under voucher number Echinodermata 021831 (www.floridamuseum.ufl.edu, John D. Slapcinsky, slapcin@flmnh.ufl.edu). Genomic DNA was extracted from the sample using a DNeasy Blood and Tissue kit (Qiagen, Valencia, CA). DNA libraries were constructed using Illumina HiSeq (Illumina, San Diego, CA), and were sequenced using HiSeq Platform, with 250-bp paired-end reads at the Hubbard Center for Genome Studies at the University of New Hampshire (Durham, NH). The DNA fragments were assembled using *de novo* assembly methods in Geneious Prime V. 2021.0.3 (https://www.geneious.com). To infer phylogenetic placement, a maximum-likelihood phylogenetic tree was constructed using MEGA-X (Kumar et al. 2018) with 1000 bootstrap replicates (Figure 1). The mitochondrial sequences used for the tree were: *Cucumaria miniata,* AY182376; *Pseudocolochirus violaceus,* NC051967; *Phyllophorella liuwutiensis*, MN198190; *Cercodemas anceps*, NC054245; *Colochirus quadrangularis,* MW218898. Annotation of the assembled genome was conducted with MITOS2 (Bernt et al. 2013).

**Figure 1.**
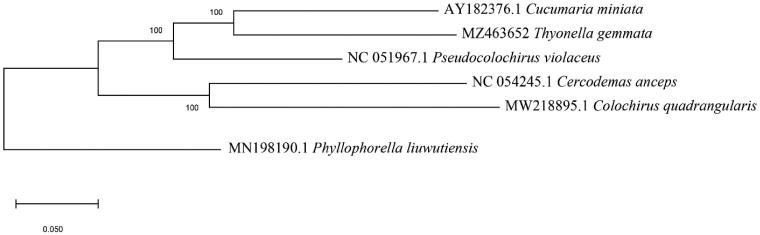
Maximum likelihood tree showing the phylogenetic relationship of *Thyonella gemmata* based on the full mitochondrial genomes of five species of sea cucumbers, with *Phyllophorella liuwutiensis* as the outgroup. Bootstrap values are shown for each node.

The complete mitogenome of *T. gemmata* was 15,696 base pairs in length (Genbank accession number: MZ463652), and the nucleotide composition was 36.10% A, 28.27% T, 23.18% C, and 12.45% G, with a total A + T content of 64.37%. There were 13 protein coding genes, 22 tRNA genes, and 2 rRNA genes within the mitogenome of *T. gemmata*.

Phylogenetic results recovered *Thyonella gemmata* in the same clade as *Cucumaria miniata* and together these are sister to *Pseudocolochirus violaceus* (Figure 1). More distantly related are the monophyletic *Colochirus quadrangularis* and *Cercodemas anceps.* Further analysis into the phylogenetic tree of *T. gemmata* showed that separated clades have distinct physiological differences. The clade consisting of *T. gemmata* and *C. miniata* have equally distributed tube feet resulting in U-shaped muscular contractions for burrowing in sediment (Pawson et al. 2010). The outgroup, *P. liuwutiensis,* has scattered tube feet with the majority of tube feet being ventrally distributed. *Phyllophorella liuwutiensis* is primarily distributed in waters in China and Korea within the Yellow Sea, where sediments are primarily composed of clay while the other two related species are primarily distributed in sandy sediment (Liao et al. 2007). This difference in sediment leads to speculation of the evolution of location and distribution of tube feet due to varying levels of sediment impermanence. This can further be applied to future phylogenetic research of holothurians to determine evolutionary relationships.

## Data Availability

The data that support the findings are openly available in NCBI at (https://www.ncbi.nlm.nih.gov/), reference number (MZ463652). The associated BioProject, SRA, and Bio-Sample numbers are PRJNA731158, SRP320569, and SAMN20122378.
